# Pu-erh Tea Extract Ameliorates Ovariectomy-Induced Osteoporosis in Rats and Suppresses Osteoclastogenesis *In Vitro*

**DOI:** 10.3389/fphar.2017.00324

**Published:** 2017-05-31

**Authors:** Titi Liu, Shihua Ding, Dan Yin, Xiangdan Cuan, Chuanqi Xie, Huanhuan Xu, Xuanjun Wang, Jun Sheng

**Affiliations:** ^1^Key Laboratory of Pu-erh Tea Science, Ministry of Education, Yunnan Agricultural UniversityKunming, China; ^2^Tea Research Center of YunnanKunming, China; ^3^College of Food Science and Technology, Yunnan Agricultural UniversityKunming, China; ^4^College of Longrun Pu-erh Tea, Yunnan Agricultural UniversityKunming, China; ^5^State Key Laboratory for Conservation and Utilization of Bio-Resources in YunnanKunming, China

**Keywords:** PTE, osteoporosis, ovariectomy, bone quality, osteoclastogenesis

## Abstract

**Background and Objective:** Tea drinking is associated with positive effects on bone health and may protect against osteoporosis, especially in elderly women. Pu-erh tea has many beneficial effects on human health; however, whether Pu-erh tea has anti-osteoporotic potential remains unclear. Thus, we investigated the effects of Pu-erh tea extract (PTE) on ovariectomy-induced osteoporosis in rats and on osteoclastogenesis *in vitro*.

**Methods:** Female Wistar rats were divided into six groups: the sham, model, and Xian-Ling-Gu-Bao capsule (XLGB) groups, and the low-, medium-, and high-dose PTE groups. Ovariectomized (OVX) rats were used as an animal model of osteoporosis. The animals were intragastrically administered distilled water, XLGB, or different concentrations of PTE for 13 weeks. Body weight, blood biochemical indicators, relative organ coefficients, femoral bone mineral density (BMD), bone biomechanical properties, and bone microarchitecture were examined and analyzed. Additionally, the *in vitro* effects of PTE on osteoclastic activities were investigated using the RAW 264.7 cell line as an osteoclast differentiation model. The effects of PTE on osteoclast differentiation and the expression of osteoclast-specific genes and proteins were determined.

**Results:** PTE reduced OVX-induced body weight gain after 6 weeks of treatment, and the high-dose exerted a significant effect. High-dose PTE significantly ameliorated OVX-induced estradiol (E_2_) deficiency. PTE treatment maintained calcium and phosphorus homeostasis and improved other blood biochemical parameters to various degrees. In addition, PTE treatment improved organ coefficients of the femur, uterus, and vagina and improved femoral BMD and bone biomechanical properties. PTE treatment strikingly ameliorated bone microarchitecture. Moreover, in the *in vitro* studies, osteoclast differentiation using the differentiation cell model was significantly inhibited by PTE without cytotoxic effects. Additionally, PTE efficaciously suppressed the expression of key osteoclast-specific genes and proteins.

**Conclusion:** PTE can ameliorate ovariectomy-induced osteoporosis in rats and suppress osteoclastogenesis *in vitro*.

## Introduction

Osteoporosis, a systemic bone metabolic disease, is caused by many factors and is characterized by decreased bone mass, bone density, and microarchitectural deterioration of bone tissue, which results in enhanced bone fragility and an increased susceptibility to fractures (Park et al., 2012; Byun et al., 2014; Roux and Briot, 2017). It has been ignored by most people because patients with osteoporosis do not have specific symptoms in the clinic, and pathogenesis is very slow (Das et al., 2004; Li et al., 2017). Currently, osteoporosis has become a serious threat to human health and eventually leads to reduced daily activity, lowered quality of life, and increased mortality (Shen et al., 2013; Tabatabaei-Malazy et al., 2017). With an increase in the aging population and life expectancy, osteoporosis has become more prevalent in older individuals worldwide (Shen et al., 2013). In particular, women are prone to develop osteoporosis after menopause because of accelerated bone turnover that is secondary to estrogen deficiency, resulting in an increased risk of fractures (Tabatabaei-Malazy et al., 2017). However, the pathogenesis of osteoporosis has not been fully elucidated. Moreover, drug treatment for osteoporosis has some limitations and may cause adverse effects in patients (Schwarz et al., 2014; Lee et al., 2016). Therefore, effective preventative measures, which involve many long-term strategies, including healthy dietary supplementation and regular physical activity, are imperative for individuals at risk of developing osteoporosis (Kleerekoper, 2006; Park et al., 2012; Michael Lewiecki, 2017).

Tea is one of the most widely consumed beverages all over the world, second only to water. Emerging studies show that tea drinking has promising protective effects on human health, and its pharmacological effects and safety have been confirmed (Xu et al., 2016). Various types of tea can be produced depending on the processing method, including green tea, black tea, and Pu-erh tea. Notably, Pu-erh tea is a distinctive Chinese tea made from crude green tea leaves by postfermentation with microorganisms. It is mainly produced in the Yunnan Province of China. Water extracts from Pu-erh tea contain abundant bioactive constituents and have shown various biological activities, including antiobesity, hypolipidemic, antidiabetic, anti-inflammatory, and anticancer properties (Yu et al., 2014; Cai et al., 2016; Wang et al., 2016).

Convincing evidence from *in vitro*, animal, and human studies using various bone loss models strongly suggests that tea drinking is closely associated with bone health and may protect against osteoporosis and osteoporotic fracture, especially in middle-aged and elderly individuals (Hegarty et al., 2000; Devine et al., 2007; Shen et al., 2013; Ng et al., 2014; Huang and Tang, 2016; Nash and Ward, 2017). Living bone is a rigid yet dynamic organ that is continuously molded, shaped, and repaired (Boyle et al., 2003). Bone homeostasis is maintained through a balance between bone formation and bone resorption. During bone remodeling, excessive osteoclastic activity and inadequate osteoblastic activity significantly contribute to osteoporosis development (Shen et al., 2013; Zeng et al., 2016). Green tea extract and bioactive components found in it, especially tea polyphenols, could effectively improve bone quality by stimulating osteoblast differentiation and suppressing osteoclast formation and differentiation (Shen et al., 2009, 2011; Oka et al., 2012; Byun et al., 2014; Vester et al., 2014). Furthermore, black tea water extract was shown to increase serum estradiol (E_2_) levels and prevent against estrogen deficiency-related osteoporotic damage in an oophorectomized rat model of osteoporosis (Das et al., 2005). However, whether Pu-erh tea has anti-osteoporotic potential has not been well elucidated.

To investigate whether Pu-erh tea extract (PTE) could be used to prevent osteoporosis, the present study was designed to evaluate the pharmacological effect of PTE in preventing osteoporosis using ovariectomized (OVX) female rats as an animal model of postmenopausal osteoporosis. Furthermore, we investigated the *in vitro* effects of PTE on osteoclastic activities using a standard osteoclast differentiation model, which will aid in understanding the molecular mechanisms underlying the protective properties of PTE against osteoporosis.

## Materials and Methods

### Reagents and Antibodies

Fermented Pu-erh tea was kindly provided by the China Academy of Pu-erh Tea Research (Yu et al., 2014; Cai et al., 2016). *E. coli*-derived recombinant mouse RANKL was obtained from R&D Systems (Minneapolis, MN, United States) and dissolved in 0.1% bovine serum albumin (BSA) in phosphate-buffered saline (PBS). Ten percent neutral formaldehyde was obtained from Jinan Biological Technology Co., Ltd, China. Xian-Ling-Gu-Bao (XLGB) capsules were purchased from Guizhou Tongjitang Pharmaceutical Co., Ltd, China. Alkaline phosphatase (ALP), calcium, and phosphorus assay kits were purchased from Zhong-Sheng BeiKong Bio-technology and Science Inc., China. Bone gla protein (BGP) and E_2_ radioimmunoassay kits were purchased from the Beijing North Institute of Biological Technology (BNIBT), China. Rat interleukin (IL)-1β and IL-6 ELISA kits were purchased from Neobioscience Biological Technology Co., Ltd, China. 3-(4,5-Dimethylthiazol-2-yl)-2,5-diphenyltetrazolium bromide (MTT) was purchased from Sigma–Aldrich (St. Louis, MO, United States). The acid phosphatase (ACP) assay kit and tartrate-resistant acid phosphatase (TRAP) staining kit were obtained from Nanjing Jiancheng Bioengineering Institute (Nanjing, China). Anti-NFATc1, anti-c-Src, anti-cathepsin K, and anti-c-Fos antibodies were purchased from Santa Cruz Biotechnology (Santa Cruz, CA, United States). Anti-β-tubulin and horseradish peroxidase-conjugated secondary antibodies were purchased from Proteintech Group, Inc. (Rosemont, IL, United States) and Thermo Fisher Scientific (Waltham, MA, United States), respectively.

### Preparation of Pu-erh Tea Extract

Pu-erh tea extract was prepared as previously described (Zhao H. et al., 2011; Zhao L. et al., 2011; Xie et al., 2017). Briefly, the required fermented Pu-erh tea amounts were boiled in water for 30 min three times. The decoction was then collected, concentrated and spray-dried. The PTE powder was dissolved in distilled water, and the pH was adjusted to 7.4 with 1 M NaOH. Analysis of the chemical composition of PTE found that caffeine, tea saccharide, and especially polyphenol were the major components, accounted for 4.18, 9.31, and 33.13%, respectively. Tea pigments [also regarded as oxidative tea polyphenols (OTP)], including theaflavins, thearubigins, and theabrownins, were abundant in the fermented Pu-erh tea (Zhao L. et al., 2011; Xie et al., 2017). Moreover, OTP were the major bioactive constituents in the PTE (Huang et al., 2014; Shan et al., 2016; Wang et al., 2016; Lu et al., 2017).

### Animals

Healthy specific-pathogen-free (SPF) female Wistar rats [12 weeks old, Cat No. SCXK-(Ji)2007-0003] from the Laboratory Animal Center of Jilin University were used in the animal experiments. All rats were housed in polypropylene cages with sterile paddy husk and were maintained under a controlled environment (humidity 50–60%, ambient temperature 24 ± 1°C, 12 h light/dark cycle). During this study, OVX rats were pair-fed a normal diet based on the average weekly food consumption of the sham control group. All experimental procedures were performed in accordance with the guidelines of the Yunnan Agricultural University Committee for Care and Use of Laboratory Animals and were approved by the Animal Experiments Ethics Committee of Yunnan Agricultural University.

### Group Designations and Treatment Administration

After 1 week of feeding adaptation, all rats, which were anesthetized with chloral hydrate, were either sham-operated (sham, *n* = 12) or ovariectomized (OVX, *n* = 70). The OVX rats were randomly divided into the model group (model, *n* = 14), XLGB capsule group (XLGB, *n* = 14; 240 mg/kg body weight/day), low-dose PTE group (low-dose, *n* = 14; 120 mg/kg body weight/day), medium-dose PTE group (medium-dose, *n* = 14; 370 mg/kg body weight/day), and high-dose PTE group (high-dose, *n* = 14; 500 mg/kg body weight/day). The animals were monitored for 15 days before initiating the therapeutic regimen to allow them to recover from the operation. The animals were continuously administered their respective treatments via intragastric administration (10 mL/kg body weight) for 13 weeks, and an equal volume of distilled water was intragastrically administered to the sham and model groups. XLGB capsules, a traditional Chinese medicine, are popularly used for the treatment of osteoporosis (Dai et al., 2013; Gao et al., 2013). The dosage of XLGB capsules for rats in this study was determined based on the dosage used in clinical trials and calculated using the dose conversion table between human and rats; additionally, the different dosage levels of PTE for rats in this study was determined and calculated according to the LD_50_ in mice and the daily recommended dose of PTE. The animals were weighed weekly throughout the treatment period. After the treatment period, rats were euthanized by deep ether anesthesia. Blood samples were obtained for biochemical analyses. The left/right femur, uterus, and vagina were collected, and the samples to be used for histological analysis were maintained in 10% neutral formaldehyde. The fixed samples were maintained at room temperature for later use.

### Biochemical Parameter Analysis of Blood Samples

The blood was centrifuged at 3,000 rpm for 10 min at 4°C after incubation at room temperature for 2 h. The serum was collected and stored at -20°C for further biochemical analysis. According to the kit instructions, ALP, ACP, calcium, phosphorus, BGP, and E_2_ levels in rat serum were determined. In addition, IL-1β and IL-6 levels in rat plasma were measured.

### Determination of Organ Coefficients

The left femur, uterus, and vagina were completely separated and weighed. Then, the organ coefficients were calculated as follows: organ coefficient of the wet or dry femur = wet or dry weight of the femur/body weight; organ coefficient of the uterus or vagina = wet weight of the uterus or vagina/body weight.

### Detection of Bone Mineral Density and Biomechanical Testing

The complete left femur of the rat was collected, and muscle and connective tissue was peeled off before analysis. Femoral bone mineral density (BMD) was rapidly determined using dual-energy X-ray absorptiometry (DEXA) according to the manufacturer’s instructions (Chunxiao et al., 2017). Briefly, the left femur was scanned, and the femoral BMD value was automatically measured.

The biomechanical properties of the left femur in OVX rats were evaluated by the three-point bending flexural test method (Gao et al., 2013; Chunxiao et al., 2017). Briefly, the femur was placed in a biomechanical testing instrument (Changchun Research Institute for Mechanical Science Co., Ltd, China). The conditions were as follows: stride distance, 20 mm; and loading velocity, 5 mm/s. The data were recorded with a computer, and the maximum deflection and maximum load were calculated.

### Uterus, Vagina, and Femur Histological Analyses

For the rat uterus, vagina, and right femur histological studies, the fresh tissues were rapidly collected, fixed in 10% neutral formaldehyde for 72 h, and removed and dehydrated before embedding. The paraffin-embedded tissues were cut into 4-μm sections, which were stained with hematoxylin and eosin (H&E) according to standard techniques as previously described (Wang et al., 2016). The uterus and vagina sections were subsequently observed under a microscope. Static images of the structures of the cortical and trabecular bone were acquired using a medical image analysis system (BI-2000, Taimeng, Chengdu Technology and Market Co., Ltd, Chengdu, China). The cortical bone thickness and trabecular bone area were measured by computer-aided software.

### Cell Culture and Maintenance

RAW264.7 murine macrophages (ATCC, Manassas, VA, United States) were used in this study. Cells were cultured in Dulbecco’s modified Eagle’s medium (DMEM) supplemented with 10% fetal bovine serum (FBS) at 37°C in a humidified atmosphere with 5% CO_2_. DMEM and FBS were obtained from Thermo Fisher Scientific and Biological Industries Israel Beit Haemek Ltd, respectively.

### Cytotoxicity Assay

To evaluate the effect of PTE on the viability of RAW264.7 cells, a cytotoxicity assay was performed using the standard MTT method (Zhang et al., 2016). Briefly, the cells were seeded onto 96-well plates at a density of 3 × 10^4^ cells/well. After overnight incubation, the cells were treated with different concentrations of PTE for 24 h. After incubating the cells with MTT solution for 4 h, the absorbance at 492 nm was detected using a FlexStation 3 Multi-Mode Microplate Reader (Molecular Devices). Cell viability was expressed as a percentage of the control.

### *In Vitro* Osteoclastogenesis Assay

To induce osteoclasts, RAW264.7 cells (2 × 10^3^ cells/well) were cultured in the presence of RANKL (50 ng/mL), XLGB (10 μg/mL), and various concentrations of PTE (20, 40, or 80 μg/mL). After 6 days, cells were fixed and then stained for TRAP activity according to the manufacturer’s protocol. TRAP-positive multinucleated cells with more than five nuclei were counted as mature osteoclasts under a light microscope (Li et al., 2011; Zeng et al., 2016).

### Quantitative Real-Time PCR (qRT-PCR) Analysis

RAW264.7 cells (1.2 × 10^5^ cells/well) were seeded in a 12-well plate and then treated with RANKL (50 ng/mL) in the absence or presence of XLGB (10 μg/mL) or PTE (20 or 40 μg/mL) for 48 h.

Total RNA was extracted by TransZol Up (TransGen Biotech, Beijing, China) according to the manufacturer’s protocol. Reverse transcription was performed using the PrimeScript RT Reagent Kit with gDNA Eraser (TaKaRa Bio, Otsu, Japan) according to the manufacturer’s protocol. qRT-PCR was performed using SYBR^®^ Premix Ex Taq^TM^ II (Tli RNaseH Plus, TaKaRa Bio), and the results were determined using a 7900HT Fast Real-Time PCR system (Applied Biosystems, Foster City, CA, United States). Data were calculated using the comparative 2^-ΔΔCT^ method, and all values were normalized to the mRNA level of the endogenous gene GAPDH (Wang et al., 2016). The primer sequences (Generay Biotech, Shanghai, China) are provided in **Table 1**.

**Table 1:** Primers used in the qRT-PCR study.

Genes	Forward (5′–3′)	Reverse (5′–3′)
GADPH	AACTTTGGCATTGTGGAAGG	ACACATTGGGGGTAGGAACA
TRAP	GCTGGAAACCATGATCACCT	GAGTTGCCACACAGCATCAC
c-Fos	CAAGCGGAGACAGATCAACTTG	TTTCCTTCTCTTTCAGCAGATTGG
c-Src	CCAGGCTGAGGAGTGGTACT	CAGCTTGCGGATCTTGTAGT
β3-Intergrin	TGACATCGAGCAGGTGAAAG	GAGTAGCAAGGCCAATGAGC
Cathepsin K	CTTCCAATACGTGCAGCAGA	TCTTCAGGGCTTTCTCGTTC
MMP-9	CGTCGTGATCCCCACTTACT	AACACACAGGGTTTGCCTTC
NFATc1	TGGAGAAGCAGAGCACAGAC	GCGGAAAGGTGGTATCTCAA


### Protein Preparation and Western Blot Analysis

RAW264.7 cells (4 × 10^5^ cells/well) were seeded in 60-mm plates and incubated overnight, and they were then treated with RANKL (50 ng/mL) in the absence or presence of XLGB (10 μg/mL) or PTE (10, 20, or 40 μg/mL) for 48 h.

Western blot analysis was performed as previously described (Cai et al., 2016). Briefly, cell lysates were prepared from cultured cells using RIPA buffer (Solarbio, Beijing, China) according to the manufacturer’s protocol. Cell extracts were normalized to determine protein concentration by the BCA method. The proteins were separated by SDS–PAGE and then transferred to PVDF membranes (EMD Millipore Corporation, Merck Life Sciences, KGaA, Darmstadt, Germany). After gentle washing, blocking, and incubation with the primary antibody, the membrane was incubated with an appropriate horseradish peroxidase-conjugated secondary antibody, and the bands were detected using a Pro-light HRP Chemiluminescent Kit (Tiangen Biotech, Beijing, China). The images were acquired with a FluorChem E System (ProteinSimple, Santa Clara, CA, United States).

### Statistical Analyses

All data are presented as the mean ± the standard errors of the mean (SEM) of the results for the samples in each experimental group or triplicate samples. Differences within groups were statistically analyzed using Student’s *t*-test. *P* < 0.05 was considered statistically significant. All analyses were performed using SPSS 17.0 (Chicago, IL, United States) and GraphPad Prism 5 (GraphPad Software, Inc., La Jolla, CA, United States).

## Results

### PTE Reduced OVX-Induced Body Weight Gain in Rats

As shown in **Table 2**, body weight gain with increasing age was observed in our study. The body weight of the model group significantly increased throughout the treatment period compared with that of the sham group (*P* < 0.01) despite the fact that the same amount of food was provided to both groups. A decreasing trend in body weight was not observed in the XLGB group compared with the model group. There were no significant differences between the PTE groups and the model group during the first 5 weeks. However, OVX rats treated with high-dose PTE exhibited rapid body weight decreases at weeks 6, 7, 8, and 13 compared with the model group (*P* < 0.05). In addition, the low- and medium-doses of PTE had a tendency to reduce the OVX-induced body weight gain after 6 weeks.

**Table 2:** Effect of Pu-erh tea extract (PTE) on body weight (g) in OVX rats^#^.

Time (week)	Sham	Model	XLGB	Low-dose	Medium-dose	High-dose
0	254.0 ± 17.5^b^	278.3 ± 22.2	279.9 ± 19.9	286.9 ± 24.1	283.9 ± 26.5	280.3 ± 25.0
1	260.3 ± 18.7^b^	290.0 ± 32.5	298.0 ± 25.6	299.1 ± 26.9	296.0 ± 26.0	288.2 ± 31.2
2	263.3 ± 18.9^c^	295.8 ± 21.2	304.4 ± 29.6	307.6 ± 29.1	300.9 ± 35.0	291.6 ± 29.6
3	268.6 ± 17.6^c^	311.5 ± 23.6	318.0 ± 27.9	312.9 ± 32.6	317.7 ± 44.0	301.9 ± 28.0
4	271.8 ± 17.8^c^	317.5 ± 22.6	322.2 ± 27.3	315.6 ± 35.7	319.1 ± 47.9	301.9 ± 29.1
5	275.1 ± 16.1^c^	327.5 ± 17.2	331.9 ± 29.1	336.1 ± 30.1	325.8 ± 38.8	311.8 ± 28.5
6	270.3 ± 14.2^c^	335.9 ± 14.8	331.0 ± 29.6	327.4 ± 33.5	325.7 ± 36.9	312.3 ± 27.0^a^
7	278.6 ± 15.6^c^	343.7 ± 15.2	336.4 ± 26.4	333.3 ± 34.0	336.0 ± 35.3	318.9 ± 36.6^b^
8	283.1 ± 24.3^c^	353.1 ± 16.6	338.7 ± 31.1	341.5 ± 36.0	343.0 ± 39.1	328.4 ± 29.5^a^
9	281.0 ± 24.3^c^	353.6 ± 15.3	349.9 ± 28.3	345.4 ± 36.3	348.8 ± 38.7	335.6 ± 30.6
10	284.9 ± 15.5^c^	351.3 ± 18.2	351.4 ± 28.4	351.3 ± 33.2	348.0 ± 40.6	332.6 ± 31.2
11	298.6 ± 16.3^c^	352.8 ± 32.7	362.5 ± 30.8	352.8 ± 34.0	351.5 ± 48.2	340.8 ± 31.4
12	296.5 ± 19.7^c^	365.9 ± 19.2	366.1 ± 30.8	368.5 ± 36.0	363.4 ± 34.8	348.3 ± 33.2
13	303.0 ± 20.8^c^	367.0 ± 17.7	366.7 ± 31.1	370.6 ± 36.2	363.9 ± 42.0	345.4 ± 33.1^a^


*^#^All data are presented as the mean ± SEM (*n* = 12–14). ^a^*P* < 0.05, ^b^*P* < 0.01, and ^c^*P* < 0.001 versus the model group.*

### PTE Ameliorated Blood Biochemical Indicators in OVX Rats

**FIGURE 1:**
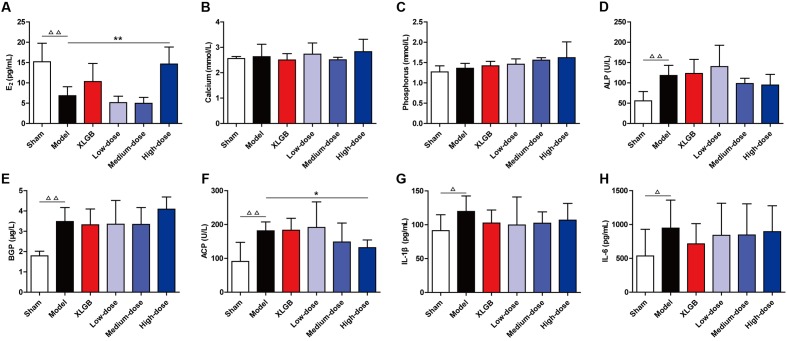
Pu-erh tea extract (PTE) ameliorated blood biochemical indicators in OVX rats. Serum E_2_ **(A)**, calcium **(B)**, phosphorus **(C)**, alkaline phosphatase (ALP) **(D)**, bone gla protein (BGP) **(E)**, and acid phosphatase (ACP) **(F)**, as well as plasma IL-1β **(G)** and IL-6 **(H)** concentrations in each group. All data are presented as the mean ± SEM (*n* = 10). ^Δ^ *P* < 0.05 and ^ΔΔ^ *P* < 0.01 versus the sham group; and ^∗^ *P* < 0.05 and ^∗∗^ *P* < 0.01 versus the model group.

To examine the effects of PTE on blood biochemical markers in OVX rats, serum E_2_, calcium, phosphorus, ALP, BGP, ACP, and plasma IL-1β and IL-6 levels were determined using the appropriate assay kits (**Figure 1**).

The level of serum E_2_ in the model group was significantly lower than that in the sham group (*P* < 0.01). Compared with the model group, OVX rats treated with XLGB had moderately increased E_2_ levels. There were no differences in the E_2_ level between the low- and medium-dose PTE groups and the model group. However, treatment of OVX rats with PTE at a high-dose significantly increased E_2_ levels compared with those in the model group (*P* < 0.01; **Figure 1A**). These findings suggested that E_2_ levels in OVX rats could be significantly improved and could reach normal levels with high-dose PTE treatment. Additionally, high-dose PTE was more effective than the drug XLGB in elevating E_2_ levels in OVX rats.

There were no differences in calcium and phosphorus levels between the model and sham groups (**Figures 1B,C**). Differences in calcium levels were not observed between the XLGB and model groups. OVX rats treated with PTE at low- and high-doses appeared to have increased calcium levels compared with the model group, but the changes were not significant (**Figure 1B**). Similarly, differences were not observed in phosphorus levels between the XLGB and model groups. Treatment of OVX rats with PTE increased phosphorus levels in a dose-dependent manner compared with the model group (**Figure 1C**). These results indicated that treatment of OVX rats with PTE could maintain calcium and phosphorus homeostasis in these animals, although significant differences were not observed between the different groups.

Additionally, we evaluated the serum biomarkers of bone formation (ALP and BGP) and bone resorption (ACP) (**Figures 1D–F**). These parameters in the model group were significantly increased compared with those in the sham group (*P* < 0.01). OVX rats treated with PTE at medium- and high-doses appeared to have lower ALP levels than did those in the model group (**Figure 1D**). In OVX rats treated with XLGB and in OVX rats treated with PTE at low- and medium-doses, BGP levels were lower than those in the model group (**Figure 1E**). Notably, treatment with PTE at a high-dose significantly prevented OVX-induced increases in the ACP level (*P* < 0.05; **Figure 1F**). Bone resorption is associated with many cytokines, including IL-1β and IL-6. There were significant differences in IL-1β and IL-6 levels between the model and sham groups (*P* < 0.05; **Figures 1G,H**). We detected lower levels of IL-1β and IL-6 in the plasma of OVX rats with XLGB or PTE treatment at any dose than in the model group. These results revealed that PTE could mildly improve bone homeostasis in OVX rats.

### Effects of PTE on the Femur, Uterus, and Vagina Organ Coefficients in OVX Rats

**FIGURE 2:**
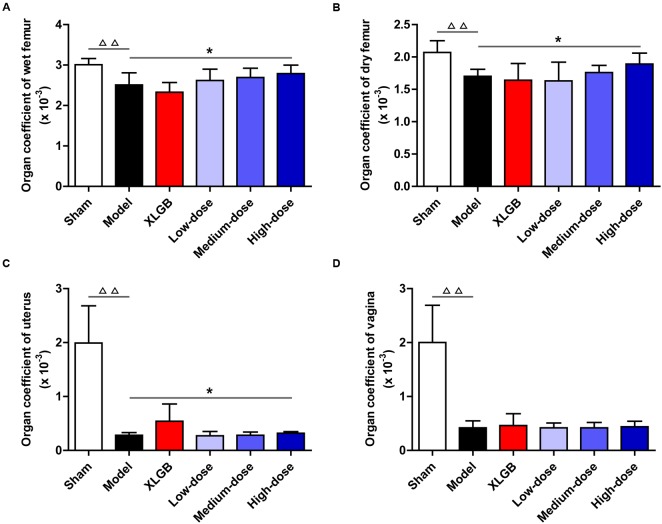
Pu-erh tea extract improved organ coefficients in OVX rats. Organ coefficients of the wet femur **(A)**, dry femur **(B)**, uterus **(C)**, and vagina **(D)** in each group. All data are presented as the mean ± SEM (*n* = 10). ^ΔΔ^ *P* < 0.01 versus the sham group; and ^∗^*P* < 0.05 versus the model group.

To further investigate the effects of PTE on osteoporosis, the femur, uterus, and vagina organ coefficients in OVX rats were obtained (**Figures 2A–D**). Compared with the sham group, the organ coefficients of the femur, uterus, and vagina in the model group all significantly decreased (*P* < 0.01). Interestingly, compared with the model group, the OVX rats treated with PTE increased the wet femur organ coefficient in a dose-dependent manner, and the high-dose PTE treatment led to a significant difference (*P* < 0.05; **Figure 2A**). A similar pattern was observed in the dry femur organ coefficient between the high- and medium-dose PTE groups and the model group (*P* < 0.05 at the high-dose; **Figure 2B**). However, no difference was observed in the organ coefficient of the uterus between almost all groups of OVX rats that underwent different treatments, except that a significant increase was observed in the high-dose PTE group compared with the model group (*P* < 0.05; **Figure 2C**). There were no significant differences in the organ coefficients of the vagina between the different treatment groups (**Figure 2D**). In addition, to investigate whether PTE protected the uterus and vagina from OVX-induced atrophy, we performed histological analyses of both tissues. H&E staining showed that PTE treatment ameliorated uterus and vagina atrophy in OVX rats, but this effect was not obvious.

### PTE Improved Femoral BMD, Biomechanical Properties, and Bone Microarchitecture in OVX Rats

**FIGURE 3:**
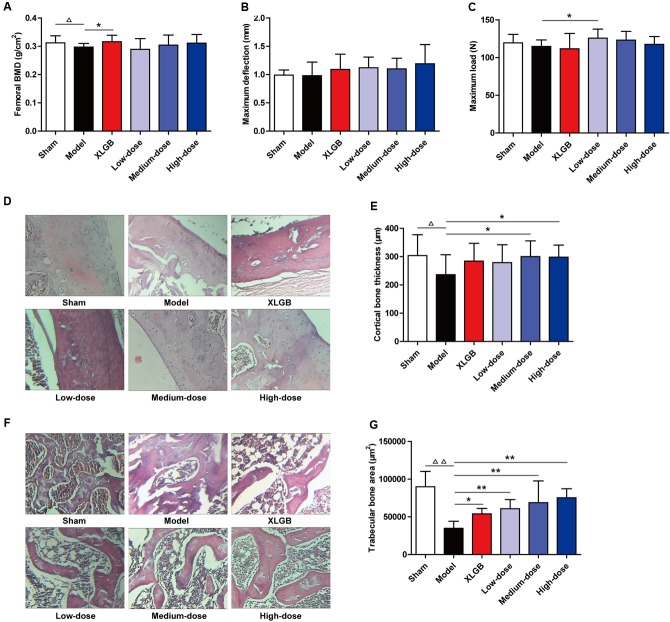
Pu-erh tea extract preserved bone quality in OVX rats. Femoral bone mineral density (BMD) **(A)**, maximum deflection **(B)**, and maximum load **(C)** in each group. Cortical bone tissue was stained with hematoxylin and eosin (H&E) **(D)**, and cortical bone thickness was calculated **(E)**; and trabecular bone tissue was stained with H&E **(F)**, and the trabecular bone area was calculated **(G)**. Representative images were acquired using a medical image analysis system, and the original magnification was ×400. All data are presented as the mean ± SEM (*n* = 10). ^Δ^ *P* < 0.05 and ^ΔΔ^ *P* < 0.01 versus the sham group; and ^∗^ *P* < 0.05 and ^∗∗^ *P* < 0.01 versus the model group.

To further identify the osteoprotective effect of PTE, the BMD, biomechanical properties, and bone microarchitecture of the femur in OVX rats were determined (**Figure 3**). The femoral BMD of the OVX rats significantly decreased to 0.297 ± 0.013 g/cm^2^, compared with 0.312 ± 0.025 g/cm^2^ in the sham rats (*P* < 0.05), indicating that ovariectomy decreased the rat femoral BMD by 4.8% after 15 weeks. Compared with the model group, the decreased femoral BMD was significantly reversed (*P* < 0.05) by XLGB administration. Compared with the model group, OVX rats treated with PTE seemed to increase femoral BMD in a dose-dependent manner, but significant differences were not observed (**Figure 3A**).

Regarding the femoral biomechanical properties (maximum deflection and maximum load), there were no significant differences between the model and sham groups (**Figures 3B,C**). Compared with the model group, OVX rats treated with XLGB and PTE showed elevated maximum deflection, but no significant differences were observed (**Figure 3B**). However, compared with the model group, OVX rats treated with PTE exhibited an increased maximum load, which was significant with the low-dose PTE treatment (*P* < 0.05; **Figure 3C**).

The above results suggest that PTE treatment could improve femoral BMD and biomechanical properties in OVX rats. To substantiate this finding further, we measured the cortical and trabecular bone microarchitectures and analyzed the cortical bone and trabecular bone parameters in the femur. Unsurprisingly, the cortical bone thickness in the model group was significantly decreased compared with that in the sham group (*P* < 0.05). Interestingly, PTE treatment effectively increased the cortical bone thickness compared with that in the model group, and the medium- and high-dose PTE groups exhibited significant differences (*P* < 0.05; **Figures 3D,E**). Similarly, the trabecular bone area in the model group was significantly decreased compared with that in the sham group (*P* < 0.01), whereas XLGB and PTE treatments significantly reversed trabecular bone loss in OVX rats (*P* < 0.05 or *P* < 0.01; **Figures 3F,G**). Notably, the treatment of OVX rats with PTE significantly increased the trabecular bone area in a dose-dependent manner compared with the model group (*P* < 0.01). These results suggested that PTE had a protective effect on the bone quality of OVX rats.

### PTE Inhibited Osteoclast Differentiation in the RAW264.7 Cell Line

**FIGURE 4:**
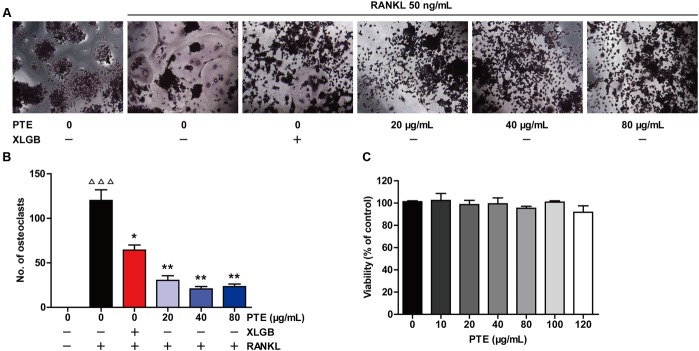
Pu-erh tea extract inhibited osteoclast differentiation in the RAW264.7 cells. RAW264.7 cells were cultured for 6 days with RANKL (50 ng/mL) in the presence of Xian-Ling-Gu-Bao (XLGB) (10 μg/mL) or the indicated concentrations of PTE and then stained for TRAP **(A)**. TRAP-positive multinucleated cells with more than five nuclei were considered mature osteoclasts, as observed under a light microscope **(B)**. The effect of PTE on the viability of RAW264.7 cells as determined by the MTT assay **(C)**. Representative images were acquired under a light microscope (magnification ×200). Values are shown as the mean ± SEM of three independent experiments. ^ΔΔΔ^ *P* < 0.001 versus the control; and ^∗^*P* < 0.05 and ^∗∗^*P* < 0.01 versus only RANKL-treated cells.

To investigate the effect of PTE on osteoclastogenesis, a standard *in vitro* osteoclast differentiation model was used. We incubated RANKL-treated RAW264.7 cells with XLGB or various concentrations of PTE and then evaluated the formation of osteoclasts using TRAP staining. TRAP-positive multinucleated cells were formed in response to RANKL stimulation, and XLGB or PTE treatment significantly reduced the number of RANKL-induced osteoclasts (*P* < 0.05 or *P* < 0.01; **Figures 4A,B**). To determine whether the inhibition of osteoclastogenesis by PTE is due to cytotoxicity, we further examined the cytotoxicity of PTE in RAW264.7 cells using the MTT assay (**Figure 4C**). As expected, PTE did not have a cytotoxic effect on osteoclast precursor cells at the concentrations used in this study. These results suggested that PTE inhibited RANKL-induced osteoclast differentiation in RAW264.7 cells.

### PTE Suppressed RANKL-Induced Osteoclast-Specific Gene and Protein Expression

**FIGURE 5:**
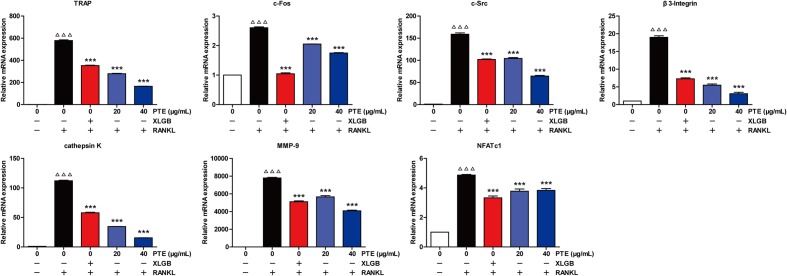
Pu-erh tea extract suppressed the RANKL-induced mRNA expression of osteoclast-specific genes. Values are shown as the mean ± SEM of three independent experiments. ^ΔΔΔ^ *P* < 0.001 versus the control; and ^∗∗∗^ *P* < 0.001 versus only RANKL-treated cells.

**FIGURE 6:**
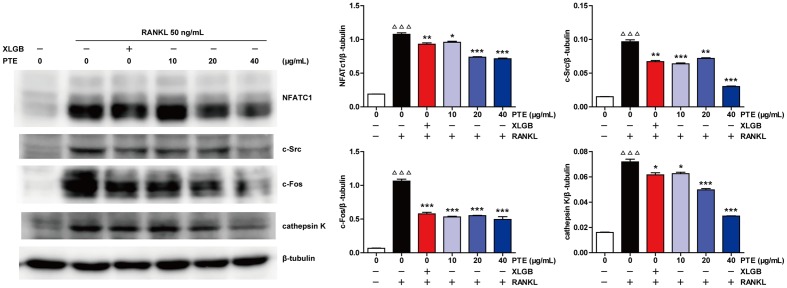
Pu-erh tea extract inhibited the expression of the RANKL-induced osteoclast-specific proteins. NFATc1, c-Src, c-Fos, cathepsin K, and β-tubulin levels were detected by western blotting, and the results were quantified using AlphaView software. Representative images are displayed. Values are shown as the mean ± SEM of three independent experiments. ^ΔΔΔ^ *P* < 0.001 versus the control; and ^∗^ *P* < 0.05, ^∗∗^*P* < 0.01, and ^∗∗∗^*P* < 0.001 versus only RANKL-treated cells.

To further examine the effect of PTE on osteoclast differentiation in RAW264.7 cells, we evaluated the expression of RANKL-induced osteoclast-specific gene markers at the mRNA level in the absence or presence of XLGB (10 μg/mL) or PTE (20 or 40 μg/mL). As shown in **Figure 5**, the mRNA expression levels of TRAP, c-Fos, c-Src, β3-Integrin, cathepsin K, matrix metalloproteinase-9 (MMP-9), and NFATc1 were significantly suppressed by XLGB or PTE during osteoclastogenesis (*P* < 0.001). Furthermore, we detected the expression of key osteoclast-specific protein markers in the absence or presence of XLGB or various concentrations of PTE. We found that XLGB or PTE treatment could significantly down-regulate the protein expression of NFATc1, c-Src, c-Fos, and cathepsin K during osteoclastogenesis (*P* < 0.05, *P* < 0.01, or *P* < 0.001; **Figure 6**), which was consistent with the results of the qRT-PCR analysis. These results strongly revealed that PTE could inhibit osteoclastogenesis.

## Discussion

Pu-erh tea, a postfermented tea that was originally grown and produced in southern Yunnan, China, is widely consumed in southeastern Asia because it has been confirmed to possess many health benefits, including weight loss, lipid metabolism regulation, cardiovascular disease alleviation, and nervous system protection (Hou et al., 2009; Kubota et al., 2011; Zeng et al., 2015; Cai et al., 2016; Li et al., 2016). Numerous studies have demonstrated that tea drinking is associated with positive effects on bone health, especially in elderly women (Hegarty et al., 2000; Devine et al., 2007; Conforti et al., 2012; Faucher, 2012; Huang and Tang, 2016). However, the potential bone protective effects of Pu-erh tea remain unclear. The OVX rat is the optimal animal model for the study of postmenopausal osteoporosis in women and is recommended by the US Food and Drug Administration and the World Health Organization (Hartke, 1999; Pan et al., 2007). In the present study, we systematically investigated the effects of PTE on ovariectomy-induced osteoporosis in rats and further investigated the *in vitro* effects of PTE on osteoclastic activities using RAW264.7 cells. Intriguingly, our results indicated that PTE could ameliorate osteoporosis *in vivo* and *in vitro*.

Unambiguously, body weight gain is a common phenomenon observed in OVX rats, and postmenopausal women are associated with a tendency to gain weight (Lee et al., 2016). In this study, the body weight of the model group was significantly higher than that of the sham group (*P* < 0.01; **Table 2**). At the beginning of the treatment, PTE treatment did not show an effect on lowering body weight. However, PTE reduced OVX-induced body weight gain in rats only after 6 weeks of treatment, suggesting that long-term tea drinking has a positive effect on weight loss, which is consistent with the results of previous studies (Zou et al., 2012). Notably, osteoporosis results from an imbalance related to faster bone resorption than bone formation. Therefore, we further investigated the biochemical markers for bone formation and resorption in blood samples (**Figure 1**). Menopause is closely associated with estrogen deficiency, which leads to decreased calcium absorption capacity in the small intestines that may accelerate the pathogenesis of osteoporosis (Das et al., 2013). In our study, we observed that the administration of high-dose PTE significantly increased serum E_2_ levels compared with those in the model group (*P* < 0.01). Previous studies have reported that high caffeine intake is a risk factor for increased calcium excretion in postmenopausal women (Macedo et al., 2015; Costa et al., 2016). Although Pu-erh tea has a high caffeine content (Huang et al., 2014), PTE could maintain calcium and phosphorus homeostasis in OVX rats. Our previous study demonstrated that the absorption of caffeine can be partly inhibited by OTP in Pu-erh tea (Huang et al., 2014), which may explain why PTE did not accelerate calcium and phosphorus loss. Moreover, PTE could lower ALP, BGP, ACP, IL-1β, and IL-6 levels in OVX rats depending on the dosage administered. Overall, PTE can ameliorate these blood biochemical indicators in OVX rats and prevent the deterioration of osteoporosis.

Importantly, *in vivo* treatment with PTE, especially at the high-dose, effectively increased the organ coefficients of the femur, uterus, and vagina in OVX rats (**Figure 2**), suggesting that PTE has protective effects on femoral, uterine, and vaginal tissues. As previously described, osteoporosis is a type of systemic skeletal disease that is characterized by bone loss and microstructure degradation of bone tissue accompanied by increased bone fragility and fracture susceptibility (Tabatabaei-Malazy et al., 2017). Femoral BMD, biomechanical properties (maximum deflection and maximum load), and bone microarchitecture are commonly used to evaluate bone quality. Our results showed that PTE treatment improved the femoral BMD and biomechanical parameters in OVX rats compared with the model group (**Figures 3A–C**). Treatment with PTE ameliorated bone microarchitecture in OVX rats due to increased cortical bone thickness and trabecular bone area in the femur (**Figures 3D–G**). These results implied that PTE could exert protective effects on bone quality. Previous studies have reported that there are many chemical components contained in the fermented Pu-erh tea, including flavan-3-ols and their derivatives, flavones and their derivatives, other phenolic compounds, alkaloids and their derivatives, and other compounds and trace elements (Lv et al., 2013). After the post-fermentation process, the caffeine, polysaccharide, and tea pigment (theaflavin, thearubigin, and theabrownin) levels were substantially increased in Pu-erh tea (Zhao L. et al., 2011; Lv et al., 2013). Notably, tea polyphenols have undergone a series of oxidative, condensing and degradative chemical processes to form oxidized tea polyphenols, which have many positive effects on human health (Lv et al., 2013; Huang et al., 2014; Shan et al., 2016; Wang et al., 2016; Lu et al., 2017). Because of the extraordinary complexity of its components, the bioactive constituents of Pu-erh tea are largely unknown. Therefore, further study is urgently required to investigate the bioactive components responsible for the anti-osteoporotic effect of Pu-erh Tea.

Evidence from *in vitro* studies indicated that the bioactive constituents found in tea may benefit bone health by enhancing osteoblastogenesis and suppressing osteoclastogenesis through the regulation of related signaling pathways (Nakashima and Haneji, 2013; Shen et al., 2013; Visagie et al., 2015). The RAW 264.7 cell line has proven to be suitable for *in vitro* studies of RANKL-induced osteoclast formation (Collin-Osdoby and Osdoby, 2012). Interestingly, our *in vitro* studies showed that PTE treatment in the differentiation cell model significantly inhibited osteoclast differentiation without cytotoxic effects (**Figure 4**). It is well known that osteoclast differentiation is directed by the expression of many related marker genes, such as NFATc1, TRAP, c-Fos, c-Src, β3-Integrin, cathepsin K, and MMP-9, most of which are regulated by NFATc1 (Li et al., 2011; Zeng et al., 2016). In this study, PTE treatment was able to effectively suppress the expression of osteoclast-specific genes (**Figure 5**) and proteins (**Figure 6**). However, future studies should investigate the precise mechanisms of PTE or its bioactive constituents in the inhibition of osteoclastogenesis.

## Conclusion

Taken together, we demonstrated that PTE can ameliorate ovariectomy-induced osteoporosis in rats and suppress osteoclastogenesis *in vitro*, suggesting that PTE could be used as a promising agent in preventing and treating osteoporosis.

## Author Contributions

JS, XW, and HX conceived and designed the experiments. HX, TL, SD, DY, and XC performed the experiments. HX, TL, CX, and XW analyzed the data. JS and XW contributed reagents/materials/analysis tools. HX, TL, and XW wrote the manuscript. All authors read and approved the final manuscript.

## Conflict of Interest Statement

The authors declare that the research was conducted in the absence of any commercial or financial relationships that could be construed as a potential conflict of interest.
